# Predictors of Response and Survival to Multikinase Inhibitors in Radioiodine Resistant Differentiated Thyroid Cancer

**DOI:** 10.3390/jpm11070674

**Published:** 2021-07-18

**Authors:** Tiziana Feola, Alessia Cozzolino, Roberta Centello, Carla Pandozzi, Maria Grazia Tarsitano, Elisa Giannetta

**Affiliations:** 1 Department of Experimental Medicine “Sapienza”, University of Rome, 00161 Rome, Italy; tiziana.feola@uniroma1.it (T.F.); alessia.cozzolino@hotmail.com (A.C.); roberta.centello@uniroma1.it (R.C.); carlapandozzi@gmail.com (C.P.); mariagrazia.tarsitano@gmail.com (M.G.T.); 2 Neuroendocrinology, Neuromed Institute, IRCCS, 86077 Pozzilli, Italy

**Keywords:** multikinase inhibitors, sorafenib, lenvatinib, differentiated thyroid cancer, radioiodine resistance, predictive marker, predictors, response to treatment, survival

## Abstract

Sorafenib and lenvatinib are the only multikinase inhibitors (MKIs) approved for the treatment of radioactive iodine refractory differentiated thyroid cancer (RR-DTC). Although they have been demonstrated to improve progression free survival and overall response rate, the risk of toxicities is very high, worsening patients’ quality of life. Therefore, predicting MKI treatment outcomes in the setting of RR-DTC is very challenging for optimizing patients’ management. The current review provides an overview of the predictive factors for the response and survival of sorafenib and lenvatinib in RR-DTC. In this setting, a systemic therapy should be considered after conducting a multidisciplinary discussion aimed at evaluating the risk-benefit ratio of the treatment and taking into account several clinical, biochemical, and molecular factors. Age, performance status, and cancer-related symptoms are the most important clinical markers to be considered prior to starting MKI treatment, together with tumor burden. Some tissue and circulating biomarkers have been investigated, those involved in the angiogenic pathways being the most promising. Finally, prospective clinical trials aimed at evaluating predictive markers for therapeutic response are needed for tailoring patient management and allowing more appropriate treatment choices.

## 1. Introduction

The differentiated thyroid cancer (DTC) represents the most common type (>90%) of tumor originating from the follicular epithelium, including the papillary and the follicular histotypes [1]. The majority of DTC can be successfully treated by thyroidectomy, radioactive iodine (RAI) therapy, and thyroid stimulating hormone (TSH)-suppressive therapy, with L-thyroxine showing favorable prognosis [2]. Only less than 5% of all cases, but 60–70% of metastatic DTC, lose the ability to uptake and concentrate RAI and to produce tireoglobulin (Tg), becoming RAI refractory (RR)-DTC [3]. RR-DTC has a poor prognosis, with a 10-year survival rate less than 20% and a mean life expectancy of 3–5 years [4]. The definition of RR-DTC is still debated, but current guidelines include four categories: (1) the absence of RAI uptake in all lesions on scintigraphy; (2) the absence of RAI uptake in some but not all lesions; (3) disease progression despite RAI uptake; and (4) reaching the maximum recommended activity of RAI [1].

Recently, the Food and Drug Administration (FDA) and the European Medicine Agency (EMA) approved two multikinase inhibitors (MKIs) for the treatment of RR-DTC, namely sorafenib and lenvantinib.

**Figure 1:**
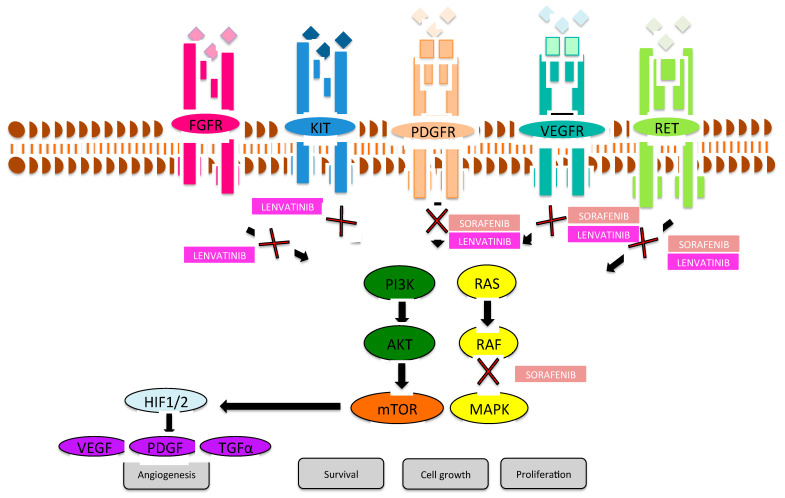
Molecular pathways of multikinase inhibitors (MKIs) in radioactive iodine refractory differentiated thyroid cancer. The MKIs (sorafenib, lenvatinib) block signaling from the tyrosine kinase receptors, preventing cell survival, growth, proliferation and angiogenesis. Abbreviations: AKT, protein kinase B; FGFR, fibroblast growth factor receptor; HIF, hypoxia-inducible factor; MAPK, mitogen activated protein kinase; mTOR, mammalian target of rapamycin; PDGFR, platelet-derived growth factor receptor; PI3K, phosphoinositide 3-kinase; RAF, rapidly accelerated fibrosarcoma kinase; RAS, rat sarcoma protein; RET, rearranged during transfection receptor protein; TGF, tumor growth factor; VEGFR, vascular endothelial growth factor receptor. The signaling pathways inhibited by the indicated drugs are represented by crossed-out arrows.

Sorafenib is an oral kinase inhibitor of vascular endothelial growth factor receptor (VEGFR)-1, -2, and -3, rearranged during transfection receptor protein (RET) -including RET/PTC-, rapidly accelerated fibrosarcoma kinase (RAF) -including BRAF V600E-, and platelet-derived growth factor receptor (PDGFR) beta (Figure 1).

For this reason, given the effectiveness of sorafenib on the RAF-1 serine/threonine kinase, in preclinical and clinical models sorafenib was experimented in inhibiting the growth of anaplastic thyroid cancer (ATC) [5,6]. Lenvatinib is an oral inhibitor of VEGFR-1, -2, and -3, fibroblast growth factor receptor (FGFR) 1 through 4, PDGFR alfa, RET, and KIT (Figure 1). Lenvatinib was also administered for ATC [7], given the multitarget effect oriented on molecular basis [8]. Sorafenib and lenvatinib showed significant improvements in progression free survival (PFS) and overall response rate (ORR) in patients with progressive RR-DTC (compared to placebo) in the DECISION and the SELECT trials, respectively [9,10]. Although they provide new therapeutic strategies against RR-DTC, unfortunately the high risk of toxicities could impair patients’ quality of life [9,10].

Therefore, the clinical management of RR-DTC is challenging and the choice of whether and when to start a target therapy should be performed in a multidisciplinary setting by an expert panel. In this context there is a growing need to understand how to predict MKI treatment response to better define which patient could benefit more from this kind of therapy.

To address this issue, we performed a review assessing the predictors of MKI clinical benefit in the setting of progressive RR-DTC, aiming at personalizing patients’ management.

## 2. Materials and Methods

We performed a keyword based PUBMED search, using relevant keywords [(predictive OR marker OR biomarker) AND (sorafenib OR lenvantinib) AND (differentiated thyroid cancer)]. The search was last updated on April 2021, and only English language studies were considered. Titles and abstracts have been screened for articles selection, identifying only those that dealt with potentially relevant factors predicting treatment outcome with sorafenib or lenvatinib in progressive RR-DTC. The selected abstracts were further assessed for a full-text evaluation. Finally, 21 papers (7 sorafenib; 14 lenvatinib) were included in the review. Predictive factors have been divided in those predicting radiological response (RECIST criteria) and those predicting survival response (prognosis).

## 3. Sorafenib

Sorafenib is a MKI previously approved for the treatment of renal cell carcinoma (2005) and hepatocellular carcinoma (2007). It has been approved by FDA in 2013 and by EMA in 2014 for the treatment of locally recurrent or metastatic, progressive DTC.

Sorafenib inhibits multiple intracellular (c-CRAF, BRAF and mutant BRAF) and cell surface kinases (KIT, FLT- 3, RET, RET/PTC, VEGFR-1, VEGFR- 2, VEGFR- 3, and PDGFR-ß) that are involved in tumor cell signaling, angiogenesis, and apoptosis (https://www.accessdata.fda.gov/drugsatfda_docs/label/2018/021923s020lbl.pdf, accessed on 1 April 2021).

Its effectiveness and tolerability have been evaluated in a phase 3 double-blind randomized trial (DECISION NCT00984282), enrolling 417 patients with progressive RR-DTC [9]. The trial demonstrated an improvement in PFS (10.8 months vs. 5.8 months for sorafenib and placebo arms, respectively) and ORR (12% vs. 1% for the sorafenib and placebo arms, respectively), whereas there were no significant differences in the overall survival (OS) between sorafenib and placebo arms. More than 60% of patients receiving sorafenib presented adverse events (AEs) responsible of drug withdrawal or dose reduction. The most frequent reported AEs (on. The most frequent reported AE dodiarrhea, alopecia, weight loss, hypertension, rash, decreased appetite, stomatitis, nausea, pruritus, and abdominal pain. Other significant AEs included squamous cell carcinoma of the skin and hypocalcemia [9].

According to data sheet, the recommended dose and schedule is 400 mg (two 200 mg tablets) taken twice daily without food. (https://www.accessdata.fda.gov/drugsatfda_docs/label/2018/021923s020lbl.pdf, accessed on 1 April 2021).

### 3.1. Predictive Markers of Radiological Response

Four studies [11,12,13,14] investigated the role of potential predictive factors of radiological response to sorafenib (Table 1).

**Table 1:** Predictive markers of radiological response to sorafenib and lenvatinib in progressive RAI-refractory DTC. * (cycle 2), ^§^ (cycle 3), ^#^ (cycle 4-5-6), • mean ± standard deviation. DCR, disease control rate; DOR, duration of overall response; MTS, maximum tumor shrinkage; OR, objective response rate; R, responders; NR, non-responders.

First Author, Year (Ref)	Study Design (Trial Name)	N° of Patients	Median Follow-Up (Months)	Biomarker	Type of Marker	Statistical Analysis	Significance	Endpoint
**Sorafenib**								
Marotta V, 2013 [12]	Retrospective, longitudinal study	17	15.5	(1) Baseline Tg (2) Tg response (3) Baseline average SUV max (PET-FDG)	(1) Circulating (2) Circulating (3) Functional imaging	ANOVA to compare R and NR	(1) *p* < 0.001 (2) *p* < 0.01 (3) *p* = 0.001	Radiological response
Yarchoan M, 2016 [14]	Phase 2 study (NCT00654238)	40	NA	nuclear pAKT	Tissue	ANOVA to compare R and NR	*p* < 0.01	Radiological response
Kim M, 2017 [11]	Retrospective multicenter cohort study	98	12.3	Tg decrease ≥ 60%	Circulating	Subgroup analyses and Cox proportional hazard model	*p* = 0.044	Disease control duration
Marotta V, 2017 [13]	Single center study	17	17	(1) VEGFA SNPs: AA/CC genotype (2) VEGFR-2 SNPs: AA + AT genotype	Genetic	Chi-square test and Odds Ratio to compare the rate of PR between groups	(1) *p* = 0.022 (2) *p* = 0.036	Radiological response
**Lenvatinib**								
Cabanillas M.E, 2015 [15]	Open-label, single-arm, phase 2 trial (NCT00784303)	58	14	(1) Tg decrease (2) Baseline Ang2 (3) Baseline IL-10 (4) Baseline fms-related tyrosine kinase 3 ligand	Circulating	Wilcoxon signed-rank test for paired samples	(1) *p* = 0.028 *, *p* = 0.002 ^§^, *p* = <0.001 ^#^ (2) *p* = 0.034 (3) *p* = 0.032 (4) *p* = 0.041	(1) MTS (2) ORR (3) ORR (4) ORR
Robinson B, 2016 [16]	Exploratory analysis from SELECT trial (NCT0132155)	261	17.1	(1) Basal body weight (2) Baseline ECOG status (3) Baseline tumor size	(1) Clinical (2) Clinical (3) Radiological	Multivariate Cox regression model	(1) *p* = 0.035 (2) *p* = 0.007 (3) *p* < 0.001	Radiological response
Tahara M, 2017 [17]	Exploratory analysis from SELECT trial (NCT0132155)	261	17.1	(1) Baseline Ang2 (2) Baseline VEGF	Circulating	Cox proportional hazards model and Multivariate analysis	(1) *p* < 0.0001 (correlation for each), *p* interaction = 0.018 (2) *p* = 0.0082, *p* = 0.0009 (correlation)	MTS, ORR
Gianoukakis A.G, 2018 [18]	Analysis from SELECT trial (NCT0132155)	261	17.1	Disease burden	Radiological	Cox proportional hazards	NA	DOR
Lee E.K, 2019 [19]	Multicenter retrospective study (NCC2017-0162)	57	8.6 ± 7.2 •	Tumor doubling time	Radiological	Pearson Chi square test between R and NR lesions	*p* = 0.02	Radiological response

Among the circulating biomarkers, baseline Tg levels and Tg response to treatment have been widely explored. A retrospective study from Marotta and coworkers found that baseline Tg levels were significantly higher in patients who showed disease progression compared with responders. Moreover, the decrease in serum Tg levels was significantly greater in patients who achieved clinical benefit compared with non-responders [12]. The role of Tg response was confirmed in a Korean study in which patients with a longer disease control duration (in a Korean study in which patients on-responders n widely explored. A retrospective study from Marotta and c [11].

Considering the role of MAPK and AKT/PI3K pathways in the progression of DTC, Yarchoan et al. investigated the molecular tumor markers from these two pathways in a phase 2 study evaluating the effectiveness of sorafenib in the treatment of RR-DTC. The authors found that low tumor expression of nuclear phospho-AKT (pAKT) was associated with partial response (PR) to sorafenib [14].

Moreover, since sorafenib showed both anti-proliferative and anti-angiogenic effects, the possible role of germline VEGF-A and VEGFR-2 single nucleotide polymorphisms (SNPs) in predicting objective response in RR-DTC patients has been explored [13]. In the study from Marotta et al. the AA/CC genotype of the VEGF-A SNPs and the AA + AT genotype of the VEGFR-2 SNP proved statistically significant association with the achievement of PR [13].

Finally, 18F fluoro-D-glucose (18-F FDG) positron emission-tomography (PET)-computed-tomography (CT) has been suggested as a useful tool in predicting radiological response, being baseline average SUVmax significantly higher in patients who showed disease progression compared with responding subjects [12].

### 3.2. Predictive Markers of Survival

Several studies evaluated the role of prognostic factors for better PFS and OS after sorafenib treatment (Table 2).

**Table 2:** Predictive markers of survival in progressive RAI-refractory DTC treated with sorafenib or Lenvatinib. DSS, disease specific survival; OS, overall survival; PFS, progression free survival.

First Author, Year (Ref)	Study Design (Trial Name)	N° of Patients	Median Follow-Up (Months)	Biomarker	Type of Marker	Statistical Analysis	Significance	Endpoint
**Sorafenib**								
Marotta V, 2013 [12]	Retrospective, longitudinal study	17	15.5	(1) Baseline Tg (2) Tg response	Circulating	Log-rank test	(1) *p* = 0.04 (2) *p* = 0.01	PFS
Kim M, 2017 [11]	Retrospective multicenter cohort study	98	12.3	(1) Absence of disease- related symptoms (2) Lung metastasis (3) Daily maintenance dose ≥ 600 mg (4) Tg decrease ≥ 60%	(1) Clinical (2) Clinical (3) Clinical (4) Circulating	Subgroup analyses and Cox proportional hazard model	(1) *p* = 0.041 (2) *p* = 0.048 (3) *p* = 0.005 (4) *p* = 0.012	PFS
Marotta V, 2017 [13]	Single center study	17	17	(1) VEGF-A SNPs: AA/CC genotype (2) VEGFR-2 SNPs: AA + AT genotype	Genetic	Log-rank test	(1) *p* = 0.006 (2) *p* < 0.001	PFS
Capdevila J, 2019 [20]	*Post-hoc analysis* From DECISION trial (NCT00984282)	125	16.2	BRAF like gene expression profile	Tissue	Multivariate Cox proportional hazard models	BRAF like *vs.* RAS like *p* = 0.015 BRAF like *vs.* noBRAL like *p* = 0.015	PFS
Kim MJ et al., 2019 [21]	Retrospective	85	19.1	Tumor doubling time	Imaging	Cox proportional hazard model	*p* < 0.01	PFS
Oh HS, 2019 [22]	Multicenter, retrospective cohort study	98	12.3	(1) No cancer-related symptoms (2) Maximal diameter of target lesion	(1) Clinical (2) Imaging	Cox proportional hazard model	(1) *p* = 0.048 (2) *p* = 0.029	OS
**Lenvatinib**								
Cabanillas M.E, 2015 [15]	Open-label, single-arm, phase 2 trial (NCT00784303)	58	14	(1) Baseline Ang2 (2) Baseline EGF	Circulating	Univariate Cox proportional hazard models	(1) *p* = 0.011 (2) *p* = 0.033	PFS
Robinson B, 2016 [16]	Exploratory analysis from SELECT trial (NCT0132155)	261	17.1	(1) Basal body weight (2) Baseline ECOG status (3) Baseline tumor size	(1) Clinical (2) Clinical (3) Radiological	Multivariate Cox regression model	(1) *p* = 0.04 (2) *p* = 0.03 (3) *p* = 0.03	PFS
Tahara M, 2017 [17]	Exploratory analysis from SELECT trial (NCT0132155)	261	17.1	(1) Baseline Ang2 (2) Tie2	Circulating	Cox proportional hazards model and log-rank tests Multivariate analysis	(1) *p* < 0.0001 (correlation) *p* interaction = 0.018 (2) *p* = 0.038 (correlation)	PFS
Sugino K, 2018 [23]	Cohort study	29	14.7	Cancer-related symptoms	Clinical	Univariate analysis	(1) *p* < 0.02 (2) *p* < 0.01	(1) PFS (2) OS
Wirth L.J, 2018 [24]	Multicenter, double-blind SELECT trial (NCT0132155)	261	17.1	Treatment emergent hypertension	Clinical	Univariate and multivariate Cox proportional hazards models	(1) *p* < 0.01 (only univariate) (2) *p* = 0.04 (multivariate)	(1) PFS (2) OS
Tahara M, 2019 [17]	Exploratory analysis from SELECT trial (NCT0132155)	261	17.1	Dose interruption (<10% versus 10% of total treatment duration)	Clinical	Multivariate Cox regression model	*p* = 0.0004	PFS
Suzuki C, 2019 [25]	Retrospective cohort study	26	26.7	(1) Tumor related symptoms (2) Bone metastasis (3) Sum of diameters of target lesions (4) Maximum tumor diameter (5) Tumor growth slope	(1) Clinical (2) Radiological (3) Radiological (4) Radiological (5) Radiological	A stepwise Cox proportional hazards	(1) *p* < 0.01, *p* = 0.05 (2) *p* < 0.01 (3) *p* = 0.02, *p* = 0.03 4) *p* = 0.04 5) *p* = 0.03	PFS, OS
Song E, 2020 [26]	A Korean multicenter study	43	16	(1) Tumor growth slope before lenvatinib initiation (2) The sum of the largest diameters of target lesions (3) Tg doubling time	(1) Radiological (2) Radiological (3) Circulating	Univariate regression analysis	(1) *p* = 0.003 (2) *p* = 0.043 (3) *p* = 0.024	PFS
Fukuda N, 2020 [27]	Retrospective cohort study	33	15.4	Neutrophil-to-lymphocyte ratio (NLR)	Circulating	Fisher’s exact test to compare outcomes according to the NLR values at the start of treatment	*p* < 0.05	OS
Takahashi S, 2020 [28]	All-case post- Marketing Observational Study	442 DTC	12	(1) Body weight, (2) ECOG PS score (3) Tumor diameter prior to lenvatinib administration (4) Tumor invasion to the carotid artery, jugular artery, trachea, skin, or other region	(1) Clinical (2) Clinical (3) Radiological (4) Radiological	Multivariate Cox regression analyses	NA	OS
Ahmaddy F, 2021 [29]	Retrospective cohort study	22	17	Response according to mPERCIST (1) at 3 months (2) at 6 months	Functional imaging	Log rank test	(1) *p* = 0.008, *p* = 0.003 (2) *p* = 0.015, *p* = 0.001	PFS, DSS
Taylor M., 2021 [30]	Retrospective analysis of SELECT trial	248	17.1	NLR	Circulating	Cox proportional hazard model	(1) *p* < 0.001 (2) *p* = 0.029	(1) PFS (2) OS

Clinical features have been advocated as potential predictors of survival and the absence of disease-related symptoms prior to sorafenib administration was associated with a better PFS [11] and OS [22] than symptomatic disease, suggesting the need to start the treatment before the onset of a clinically relevant disease.

Moreover, clinicians should taking into account the daily sorafenib maintenance dose, being a dose ib mainteassociated with better PFS [11].

The tumor burden was demonstrated to be an important prognostic factor, and while patients with lung metastasis alone had a better prognosis [11], the maximal diameter of target lesion was significantly associated with a minimally increased risk of death [22]. Furthermore, tumor doubling time, reflecting the tumor growth rates, was associated with a worse survival outcome in terms of PFS [21].

Considering the role of the mutational status on DTC progression, there is a great interest in finding tissue biomarkers that could correlate with outcome or predict benefit from sorafenib therapy. In a post hoc exploratory RNA-seq analysis using tumor samples from patients enrolled in the DECISION trial, the RNA-expression profiles and PFS were found to significantly correlate. In the sorafenib arm, patients harboring the BRAF-like profile had a significantly better survival than those with RAS-like and NoBRaL profiles [20].

Finally, the VEGF-A and VEGFR-2 SNPs (AA/CC genotype of the VEGF-A and the AA + AT genotype of the VEGFR-2), which have been demonstrated to correlate with tumor response, were also shown to be associated with a better PFS [13].

## 4. Lenvatinib

Lenvatinib is a MKI which received FDA and EMA approvals in 2015 for the treatment of locally recurrent or metastatic progressive DTC. Lenvatinib selectively inhibits the kinase activities of VEGFR1 (FLT1), VEGFR2 (KDR), and VEGFR3 (FLT4), in addition to other proangiogenic and oncogenic pathway-related receptor tyrosine kinases, including FGFR1, 2, 3, and 4, the PDGFR alfa, KIT, and RET (https://www.accessdata.fda.gov/drugsatfda_docs/label/2018/206947s007lbl.pdf, accessed on 1 April 2021).

Its effectiveness and tolerability have been evaluated in a phase 3 randomized, double-blind, multicenter study (SELECT trial NCT01321554), including 392 patients with progressive RR-DTC, that demonstrated significant improvement in PFS and a high ORR among patients receiving treatment with lenvatinib compared with those receiving placebo [10]. The median PFS was 18.3 months in lenvatinib group and 3.6 months in placebo group. The ORR was 64.8% for patients receiving lenvatinib and 1.5% for patients receiving placebo. The incidence of treatment-emergent AEs of all grades was higher in the lenvatinib group (97.3%) compared with the placebo group (59.5%) [10]. Hypertension was the most common treatment-emergent AEs associated with lenvatinib treatment [24]. The other common any-grade AEs in lenvatinib-treated patients included proteinuria, diarrhea, fatigue, rash, and palmar-plantar erythrodysesthesia syndrome [31].

As per the data sheet recommendations, the daily dose is 24 mg once daily and it might be modified according to the dose/toxicity ratio (https://www.accessdata.fda.gov/drugsatfda_docs/label/2018/206947s007lbl.pdf, accessed on 1 April 2021).

### 4.1. Predictive Markers of Radiological Response

In the last years, several potential predictive factors of response to lenvatinib have been evaluated including clinical, radiological, and circulating markers (Table 1).

The major clinical factors have been investigated in an exploratory analysis from SELECT trial, in which baseline Eastern Cooperative Oncology Group (ECOG) performance status and body weight were found to be significantly associated with percent tumor size reduction in the multivariate model [16]. Baseline tumor size, measured by summing target lesions diameters, was also found to be a predictive factor of radiological response [16]. The role of disease burden was confirmed in another analysis from the SELECT trial, in which the median duration of response was shorter in patients with greater disease burden [18]. Moreover, in a Korean multicenter retrospective study, patients with rapidly progressive disease and a shorter initial tumor doubling time (<6 months in patient-based assessment) were more likely to respond to lenvatinib [19].

Similarly to sorafenib, Tg has been advocated as a potential circulating biomarker. An open-label, single-arm, phase 2 trial found that Tg decrease showed a statistically significant correlation with the maximum tumor shrinkage, beginning on cycle 2 and lasting at several additional assessment points [15]. In this study, circulating cytokines (low baseline levels of IL-10 and high baseline levels of fms-related tyrosine kinase 3 ligand) as well as angiogenic factors (low baseline levels of angiopoietin-2 -Ang2-) correlated with improved ORR after lenvatinib therapy [15]. The role of angiogenic factors was further investigated in an exploratory analysis from a SELECT trial in which low baseline Ang2 level was found as a predictive biomarker of maximum tumor shrinkage for patients in lenvatinib group. Although baseline Ang2 and VEGF levels correlated with ORR, neither were predictive of ORR to lenvatinib [17].

### 4.2. Predictive Markers of Survival

Several studies evaluated prognostic markers of survival during lenvatinib treatment (Table 2).

In addition to radiological response, baseline body weight and ECOG status were associated with PFS in an exploratory analysis from SELECT trial [16]. Recently, these data have been confirmed in all-case post-marketing observational study by Takahashi et al., in which in multivariate analysis both these parameters were demonstrated as baseline prognostic factors affecting OS in patients with RR-DTC [28].

Similarly to sorafenib, the presence of disease-related symptoms [23,25] and a high tumor burden [25,26,28] were associated with poorer PFS and OS. Some studies highlighted the importance of tumor rate growth as a key factor to predict the outcome of lenvatinib treatment [16,25,26]. Indeed, Song and coworkers showed that patients with faster tumor growth at baseline and after the initiation of treatment had poorer survival [26]. Conversely, Suzuki at al found no association of tumor growth at baseline with PFS, but found that tumor growth after the initiation of lenvatinib and the ratio between these two parameters were associated with PFS [25]. Accordingly, in the paper of Robinson et al., in a multivariate model the percent change in tumor size at the first radiological tumor assessment was a marginally significant positive predictor for PFS (*p* = 0.06) [16].

Other clinical features have been extrapolated from the SELECT trial and advocated as predictors of survival to lenvatinib. Specifically, treatment emergent hypertension [24] and a dose interruption <10% [17] were associated with better survival outcomes.

Recently, the role of functioning imaging with 18F-FDG PET-CT was evaluated to improve treatment personalization [29]. In the paper from Ahmaddy et al., all responders to lenvatinib (according to RECIST criteria) showed a decline in nearly all PET-parameters from baseline to the three month follow-up and from baseline to the six month follow-up. At both the three and six months follow-ups, non-responders according to mPERCIST showed significantly worse survival outcomes [29].

Among circulating factors, the role of Tg, angiogenic and immune markers have been evaluated.

Song et al. showed that Tg doubling time was associated with PFS, but the analysis was confined to 34 out of 43 patients (79%) with negative Tg antibodies [26]. In an open-label, single-arm, phase 2 trial, Cabanillas et al. found that low baseline Ang2 and high baseline epidermal growth factor (EGF) levels were correlated with survival in a univariate analysis [15]. Subsequently, an exploratory analysis from the SELECT trial confirmed the role of baseline Ang2 and Tie2 as prognostic factors [17].

Finally, in a recent study Fukuda et al. explored the role of neutrophil-to-lymphocyte ratio (NLR) as a predictor of survival to lenvatinib therapy and showed that the median OS was significantly longer in the lower NLR group (<3) than in the higher NLR group when starting lenvatinib treatment [27]. These data have been confirmed by Taylor and coworkers in a post hoc analysis from a SELECT trial in which they found that patients with a baseline NLR ≤S3 had better PFS and OS than patients with a baseline NLR > 3 [30,31]. These findings suggest that NLR could be used as an indicator for starting lenvatinib treatment.

## 5. Final Remarks

The clinical management of RR-DTC still represents a challenge to the decision concerning whether and when a target therapy should be performed in a multidisciplinary setting by an expert panel.

In the last years, there has been a growing interest in finding new biomarkers for RR-DTC therapy, which are strongly needed to customize treatment strategies.

The current review summarizes the literature evidence on potential predictors of radiological response and survival outcomes in patients with progressive RR-DTC, candidate to MKI treatment. In the setting of progressive RR-DTC, a systemic therapy should be considered after the evaluation of risk-benefit ratio of treatment and taking into account patients characteristics, tumor features, tissue, and circulating biomarkers.

### 5.1. Patients Characteristics

Clinicians should consider the age and the general status of the patients prior to starting MKI treatment. The incidence of AEs is more frequent in older than in younger patients, with a higher need of dose adjustments, although no significant differences were observed in PFS between the two groups [9,28].

The baseline ECOG score and body weight have been demonstrated to be predictive factors of radiological response as well as prognostic factors for lenvatinib treatment in an exploratory analysis from the SELECT trial. This was also confirmed in a Japanese real-world clinical setting [16,28], suggesting that patients with a good performance status could benefit more from the treatment. In accordance with these findings, an Italian real-world experience with lenvatinib showed less favorable efficacy outcomes than the registration trial, probably due to a negative selection of the study population that included patients with worse clinical features [32].

According to these findings, the absence of cancer-related symptoms is another crucial prognostic clinical factor in the decision-making process leading to the initiation of MKI treatment. Indeed, asymptomatic patients seem to benefit more from sorafenib and lenvatinib therapy [11,22,23,25], suggesting that MKIs should be started prior to the occurrence of cancer-related symptoms.

Finally, the genetic background could influence the response to MKIs. A set of SNPs of the VEGF-A and VEGFR-2 genes, which represent the most important angiogenic regulators, could be useful to predict radiological response and survival outcome of sorafenib treatment [13]. Patients harboring a genetic background associated with less efficient angiogenic mechanisms seem to be more responsive to sorafenib [13].

### 5.2. Tumor Features

The role of tumor burden in predicting MKI treatment outcome has been evaluated in several RR-DTC settings. A low tumor burden has been correlated with better tumor response to lenvatinib [16,18] and has been associated with better survival outcome [11,22]. More debated is the role of tumor rate growth, since highly proliferative tumors seem to be more responsive to lenvatinib [19]. However they have been associated with a worse prognosis [21,25,26].

Recently, the role of 18F-FDG PET-CT as a useful tool for predicting tumor response has been advocated and baseline SUVmax has been found to correlate with radiological response to sorafenib treatment [12]. Moreover, the assessment of lenvatinib response by (mPERCIST) appeared to be stronger correlated with survival outcomes than RECIST, improving treatment individualization through the selection of patients with an increased likelihood of benefit from lenvatinib [29]. These findings suggest that functional imaging should be included in the diagnostic work-up of patients with RR-DTC candidate to MKIs.

### 5.3. Tissue Biomarkers

Different tissue biomarkers have been explored as potential predictors of response to MKIs. Lower nuclear pAKT tumor expression has been associated with higher response rate to sorafenib [14]. Several genetic alterations can involve the PI3K/AKT signaling pathway, leading to DTC pathogenesis and progression. Therefore, it is possible that increased pAKT expression in tumor cells represents a mechanism of escape to sorafenib [14]. These findings suggest that a combination therapy with sorafenib and an inhibitor of the PI3K/AKT signaling pathway could be considered in RR-DTC patients.

Subgroup analyses of the DECISION and SELECT trials, evaluating the effectiveness of sorafenib and lenvatinib treatment, respectively, did not show any difference in efficacy in relation to BRAF or RAS genetic alterations. Conversely, RNA-seq analysis from DECISION trial were associated with a BRAF-like profile and a better outcome of sorafenib treatment compared with RAS-like and NoBRaL profile, suggesting that the expression profile may be useful for a better disease characterization before recommending systemic therapy with MKIs [20].

### 5.4. Circulating Biomarkers

Serum Tg is a well-recognized marker of disease after thyroidectomy, since detectable levels are correlated with persistent loco-regional or metastatic disease [33]. However, the role of baseline Tg and Tg decrease in predicting radiological response and survival to MKI treatment is a very debated and controversial issue. Low baseline Tg level [12] and a greater Tg decrease seem to be associated with a higher benefit [11,12,15,26], but some studies failed to find this association [9,11,34,35]. Moreover, during treatment, transient Tg oscillations are a frequent phenomenon that may not necessarily reflect morphologic tumor progression [36]. Long-term follow-up studies will be useful to clarify the prognostic value of Tg.

Considering the mechanism of action of MKIs, some studies have focused on the angiogenic pathways to find new biomarkers. Increased VEGF expression is significantly associated with angiogenesis and advanced-stage RR-DTC. Another molecular driver of tumor growth in DTC is FGF/FGFR. Finally, Ang2 is a regulator of angiogenesis that has been demonstrated to be a predictive marker in different cancer settings. Low Ang2 could be useful as circulating marker to select patients who will benefit more from lenvatinib treatment [17].

Recently, NLR has received a great interest as predictive marker in oncology, reflecting the anti-tumor immunity status. A lower NLR has been associated with better survival outcome to lenvatinib, providing a useful and feasible tool to select patients’ candidacy for MKI treatment [27,30].

Several factors including clinical, molecular, circulating, and tumor markers could help in selecting patients who will benefit more from MKI treatment. However, the majority of data emerged from secondary analyses of SELECT and DECISION trials or from small retrospective studies. Therefore, the lack of trials aiming at investigating predictive biomarkers of MKI effectiveness limits the clinical applicability of these findings, which should be considered as a basis for further prospective multicenter studies.

## 6. Conclusions

Nowadays, sorafenib and lenvatinib are the only MKIs which have received approval for the treatment of patients with progressive RR-DTC. Unfortunately, MKI treatment is burdened with important AEs, potentially affecting patients’ quality of life. Thus, evaluating the risk-benefit ratio is mandatory before starting treatment.

Evidence from the available literature shows that several factors have been advocated as potential predictors of response to MKIs, although none of them has been validated as an ideal biomarker.

Nevertheless, these factors could help the clinician in selecting patients with RR-DTC candidate to MKIs. Published data agree in suggesting that patient clinical status and the presence of tumor-related symptoms should be taken into account, preferring to treat those asymptomatic patients in better clinical condition. Moreover, tumor burden should also guide the choice of MKIs, with a lower burden associated with a better radiological response and survival outcome.

Prospective studies aiming at validating biomarkers predicting MKI tumor response and prognosis are strongly needed to personalize therapy of patients with progressive RR-DTC.

## Data Availability

Data sharing not applicable.
